# Editorial: Chromatin architecture in gene regulation and disease

**DOI:** 10.3389/fgene.2023.1254865

**Published:** 2023-08-18

**Authors:** Xiang Wang

**Affiliations:** Children’s Hospital of Philadelphia, Philadelphia, PA, United States

**Keywords:** chromatin architecture, human disease, genome organization, gene expression, enhancer-promoter interaction

## Introduction

Chromatin architecture, the three-dimensional organization of DNA and its associated proteins, plays a pivotal role in gene regulation and disease. Recent advances in genomics and molecular biology have shed light on the intricate relationship between chromatin architecture and various biological processes. This Research Topic aims to highlight the significance of understanding chromatin architecture in unraveling the complexities of gene regulation and its implications for human health and disease.

## Insights into the genome organization

Chromatin architecture influences gene regulation by regulating the accessibility of DNA to transcriptional machinery. It orchestrates the interactions between regulatory elements, such as enhancers, promoters, and insulators, and their target genes. Elucidating the mechanisms by which chromatin architecture modulates gene expression holds the key to understanding fundamental biological processes, including development, cellular differentiation, and environmental responses. Kyrchanova et al. review the enhancer-promoter interaction and chromosomal architecture. They discuss the difference of chromosomal architecture between mammals and *Drosophila*. Scott et al. review how changes in the mechanical environment induce alterations in chromatin architecture. They discuss the critical questions need to be addressed to give insights into the mechanically induced disease.

## Uncovering chromatin architecture during DNA replication

DNA replication is a highly regulated process that ensures the accurate duplication of genome prior to cell division. The replication timing (RT) of different regions of the genome is not uniform and has been found to correlate with chromatin architecture (Marchal et al., 2019; Pope et al., 2014). Yu et al. examine the 3D genome characteristics at the RT-switching regions. This study expands our understanding of the relationship between RT, histone modification and 3D chromatin structure in cell differentiation.

## Chromatin architecture in cancer

Aberrations in chromatin architecture have been implicated in various human diseases, including cancer, neurodegenerative disorders, and developmental abnormalities. Dysregulation of gene expression due to altered chromatin structure can disrupt delicate cellular homeostasis, leading to disease initiation and progression. Gridina and Fishman review how the organization of the cancer cell genome differs from the healthy genome at various levels. They discuss how these changes in genome organization contribute to cancer development.

Unraveling the connections between chromatin architecture and disease provides crucial insights for the development of novel diagnostic, prognostic, and therapeutic strategies. Wang et al. examine the alterations of chromatin organization in doxorubicin-resistant breast cancer and identify potential diagnostic or therapeutic targets associated with the reorganization of chromatin architecture. This study highlights the connection between 3D genome reorganization, chromatin accessibility, and gene transcription and provides valuable insights into the epigenomic mechanisms underlying doxorubicin resistance and offers potential targets for breast cancer treatment.

The study of chromatin architecture in gene regulation and disease represents a rapidly advancing field that offers unprecedented insights into the fundamental mechanisms governing cellular processes and disease pathology. By understanding the principles underlying the intricate orchestration of chromatin architecture and gene regulation, researchers can pave the way for innovative approaches to diagnose, prevent, and treat a wide range of diseases. Continued exploration of this Research Topic has the potential to redefine our understanding of human health and open new avenues for personalized medicine in the future.
